# Iodine and Selenium Intakes of Postmenopausal Women in New Zealand

**DOI:** 10.3390/nu9030254

**Published:** 2017-03-09

**Authors:** Louise Brough, Caroline A. Gunn, Janet L. Weber, Jane Coad, Ying Jin, Jasmine S. Thomson, Mathilde Mauze, Marlena C. Kruger

**Affiliations:** 1 Massey Institute of Food Science and Technology, School of Food and Nutrition, Massey University, Private Bag 11222, Palmerston North 4442, New Zealand; caroline.gunn@fonterra.com (C.A.G.); j.l.weber@massey.ac.nz (J.L.W.); j.coad@massey.ac.nz (J.C.); y.jin@massey.ac.nz (Y.J.); j.a.thomson@massey.ac.nz (J.S.T.); mathilde.mauze@etu.unilasalle.fr (M.M.); m.c.kruger@massey.ac.nz (M.C.K.); 2 Fonterra Research & Development Centre, Private Bag 11029, Dairy Farm Road, Palmerston North 4442, New Zealand

**Keywords:** iodine, selenium, postmenopausal women, iodine fortification

## Abstract

Iodine and selenium are required for thyroid function. This study investigated iodine and selenium intakes in healthy, women aged 50–70 years (*n* = 97) from three cities in the North Island of New Zealand, after mandatory fortification of bread with iodised salt. Iodine and selenium concentrations were determined in 24-h urine samples; daily intakes were extrapolated from amounts in urine (90% and 55% of daily intake, respectively). Three day diet diaries (3DDD) also estimated selenium and iodine (excluding iodised salt) intake. Median urinary iodine concentration (UIC) was 57 (41, 78) µg/L, indicating mild iodine deficiency. Estimated median iodine intake based on urine was 138 (100, 172) µg/day, below Recommended Dietary Intake (RDI) (150 µg/day) with 25% below Estimated Average Requirement (EAR) (100 µg/day). Estimated median selenium intake was 50 (36, 71) µg/day based on urine and 45 (36, 68) µg/day using 3DDD, below RDI (60 µg/day) with 49%–55% below EAR (50 µg/day). Median bread intakes were low at 1.8 (1.1, 2.7) serves/day; 25% consumed ≤1 serve/day. Although population iodine intakes improved following mandatory fortification, some had low intakes. Selenium intakes remain low. Further research should investigate thyroid function of low consumers of iodine fortified bread and/or selenium in New Zealand.

## 1. Introduction

The New Zealand food supply contains low levels of iodine and selenium [1]. Both nutrients are required for thyroid function and the thyroid gland contains the highest concentration of these minerals in the human body [2]. Thyroid hormones are required for the control of metabolic processes, growth and development, especially of the brain and central nervous system [3]. Iodine is a constituent of the thyroid hormones, thyroxine (T4) and triiodothyronine (T3), whereas selenium-dependent iodinase generates the active T3 form from the inactive T4 form [2]. Selenium is also a constituent of the antioxidant enzyme glutathione peroxidase (GPx); iodine deficiency can result in accumulation of hydrogen peroxide and concurrent selenium deficiency may impair the degradation of hydrogen peroxide leading to damage of the thyroid gland [2]. Thus selenium deficiency could potentially exacerbate the consequences of mild iodine deficiency which is present in New Zealand [4].

In Australia and New Zealand the Estimated Average Requirement (EAR) and Recommended Dietary Intake (RDI) for adult women for iodine are 100 and 150 µg/day and for selenium are 50 and 60 µg/day, respectively [5]. The EAR cut-point method can be used to assess population nutrient intake providing nutrient requirements are normally distributed (e.g., selenium and iodine); the percentage below the EAR approximates the proportion that is at risk of dietary inadequacy [6,7,8]. For a population to have a very low prevalence of inadequate dietary intakes, the mean/median intake should be above the RDI [6]. Urine samples have routinely been used to assess iodine intake and status in populations [9] and the WHO defines iodine sufficiency in an adult population as a median urinary iodine concentration (UIC) of >100 µg/L in spot urine samples, providing no more than 20% of samples are <50 µg/L [10]. A selenium intake of 20 µg/day is needed to prevent Keshan disease and intakes between 25–40 µg/day in New Zealand adults have not been associated with deficiency symptoms [11]. Duffield et al. showed a selenium intake above 30 µg/day was required for optimal deiodinase activity, and that intakes below this may alter the ratio of T4/T3 [12]. 

Sufficient levels of thyroid hormones are important during pregnancy and the first three years of life for optimal brain development, and severe deficiency can result in cretinism [10]. However, even mild iodine deficiency can have effects throughout the lifecycle and has been associated with a higher rate of multinodular toxic goitre, especially in women over 50 years of age [13]. The prevalence of thyroid disease in New Zealand women is around 5% [14] but this increases with age to 7%–14% of women over 50 years of age [15]. Historically, iodine deficiency was a problem throughout New Zealand and parts of Australia [4]; to prevent iodine deficiency the mandatory fortification of all bread (except organic) with iodised salt was introduced into both Australia and New Zealand, in September 2009 [16]. The aim of this study was to investigate iodine and selenium intakes after the mandatory fortification of bread with iodised salt in a population of postmenopausal women.

## 2. Materials and Methods

Self-selecting women were recruited into an intervention trial (The Scarborough Fair study) from Palmerston North, Hawkes Bay and Auckland, New Zealand from August 2011, the details of the study have been reported previously [17]. Ethical approval for the study was obtained from Massey University Ethics Committee (Southern A), reference number 11/11. All participants gave written informed consent for inclusion before they participated in the study. Participants were healthy, postmenopausal (≥5 years) women aged between 50–70 years. The results presented here constitute baseline data before the intervention began.

A subsample of participants collected urine over 24-h and the total volume was determined. Samples were stored without preservative at −20 °C, prior to analysis. 24-h urine samples were excluded as inaccurate due to under or over collection if urine volume was below 1 L and urinary creatinine below 5 mmol/day or extreme outliers for urinary creatinine (>±3 SD) [18]. 

Iodine and selenium concentrations in urine samples were determined by an accredited commercial laboratory (Hill Laboratories, Hamilton, New Zealand) using inductively-coupled plasma mass spectrometry [19]. Quality control procedures included analysis of blanks, analytical repeats and spiked samples to ensure accuracy and precision. Calibration standards and checks were undertaken on every run and the limit of detection was 0.001 mg/kg for iodine and 0.002 mg/kg for selenium. Creatinine was measured using the Jaffe Method Flexor E (Vital Scientific NV, 6956 AV Spankeren/Dieren, The Netherlands) by Massey University Nutrition Laboratory. 

Daily iodine and selenium intakes were estimated by extrapolation of 24-h urinary iodine and selenium excretion based on the estimated amounts excreted via urine (90% for iodine [9] and 55% for selenium [11]). The ratios of both iodine and selenium to creatinine were also calculated (µg/g).

Dietary intake was also assessed using three day diet diaries (3DDD). Participants were given verbal and written instructions on how to complete the diary and also visual examples of standard portion sizes (cups, tablespoons, etc.). All food and drink consumed over 2 weekdays and 1 weekend day was recorded together with recipes for homemade dishes and nutrition information panels from processed food packages. A New Zealand Registered Nutritionist (C.G.) reviewed the 3DDD with each participant. The 3DDD were entered into Foodworks (version 8, Xyris, Qld, Australia) and selenium intake was determined over the 3 days. Since the mandatory fortification of bread with iodised salt, the iodine content has not been determined for all commercially made bread in New Zealand. The Ministry for Primary Industries has estimated iodine concentrations for categories of bread (e.g., white, fibre white, fruited, mixed grain etc.) [20], and these were entered into Foodworks. It is difficult to quantify the amount of discretionary salt added to food, also we did not specifically ask about the use of iodised salt, so iodine intakes excluding iodised salt were determined.

The number of serves of foods which are good sources of iodine and/or selenium (bread, fish and dairy) consumed per day were determined using the Ministry of Health standard definitions [21]. A serve of bread was defined as 1 slice of bread or 1 bread roll; a serve of fish was equal to 100 g cooked fish or seafood or a medium fish fillet; and a serve of dairy was defined as 1 glass (250 mL) of milk, 1 small pot of yoghurt (125–150 g) or 2 slices (40 g) of cheese (e.g., edam).

Body weight was determined in subjects wearing light clothing and without shoes to the nearest 0.1 kg using digital scales (UWE Gilbarco, Greensboro, NC, USA). Body height was measured to the nearest 0.5 cm without shoes using a stadiometer (SECA 213). BMI was calculated using weight (kg)/height (m^2^). 

Data were analysed using IBM SPSS (version 20, Chicago, IL, USA). Normality of data were tested using Shaprio-Wilk test; parametric data were expressed as mean with standard deviation and non-parametric data as median with quartile 1 and quartile 3 (Q1, Q3; based on Weighted Average). Much of the data were non-parametric thus bivariate correlations were tested using the nonparametric Spearman’s rho correlation coefficient. Scatter plots were generated for suspected bivariate correlations and visually inspected to verify correlations. Non-parametric, related samples were compared using the Wilcoxon signed rank test. Independent non-parametric samples were compared using the Mann-Whitney U test. 

## 3. Results

A total of 148 women were recruited into the original study; 24-h urine samples were provided by a subsample of this group (*n* = 104). No samples were classified as under or over collections. A complete data set of iodine, selenium and creatinine excretion and dietary data were available for 97 participants, the data analysis presented here is based on these 97 samples only. Participants were predominantly European (97%) with only 3% identifying as Maori or Pacific Islander; mean body weight was 70.1 ± 13.2 kg, BMI 26.5 ± 5.1 kg/m^2^, age 60.3 ± 4.1 years and 11.1 ± 4.6 years since menopause. There was no difference in any of these measures for women for whom there was complete data sets compared to without (*n* = 51). The participants were not representative of the New Zealand population (74% European, 15% Maori, 7% Pacific Islander [22]), which potentially reflects the difficulty in recruiting older indigenous and ethnic minority women without significant health problems. 

One participant reported use of an iodine supplement and had an extremely high iodine excretion (estimated intake 4800 µg/day) over 4 times the upper limit (1100 µg/day). Another two participants had high selenium excretions (estimated intake 709 and 817 µg/day) around twice the upper limit (400 µg/day). As these data were non-parametric these remained in the analysis. No other participants reported taking iodine or selenium supplements. 

The median UIC for the 97 participants was 57 (41, 78) µg/L and 39% had UIC < 50 µg/L (Table 1), indicating mild iodine deficiency according to WHO criteria [10]. The estimated median iodine intake based on 24-h urine excretion was 138 (100, 172) µg/day, lower than the RDI (150 µg/day), with 25% of participants below the EAR (100 µg/day), suggesting dietary inadequacy (Table 2). Median estimated iodine intake (excluding iodised salt) based on the 3DDD was 79 (55, 97) µg/day which was significantly lower (<0.001) than estimated using 24-h urine. However, the current study iodine intake based on dietary assessment is similar to the median value of 80 (64, 101) µg/day found recently in NZ women aged 45–64 years using an FFQ (excluding iodised salt) [23]. Dietary intake of iodine based on 24-h urine (µg/day) was weakly correlated with iodine intake (excluding iodised salt) based on 3DDD (µg/day) (*p* = 0.017, *r* = 0.242) (Table A1). There was no significant difference in iodine or selenium intake based on 3DDD for participants who provided a 24-h urine sample compared to those who did not. 

Using urinary excretion the estimated median selenium intake for the current study population was 50 (36, 71) µg/day (Table 2), below the RDI (60 µg/day), with 49% below the EAR (50 µg/day), suggesting dietary inadequacy within the population. Of the participants, 11% had intakes below 30 µg/day suggested for adequate deiodinase function [12]. Median estimated selenium intake based on 3DDD was 45 µg/day (55% < EAR and 11% < 30 µg/day), not significantly different (*p* = 0.75) than estimated using 24-h urine. Selenium intake based on dietary assessment was weakly correlated with selenium excretion as both µg/day (*r* = 0.329, *p* = 0.001) and µg/L (*r* = 0.216, *p* = 0.033). Iodine and selenium excretions were weakly correlated as both µg/day (*r* = 0.385, *p* < 0.001) and µg/L (*r* = 0.385, *p* = 0.009), as were iodine and selenium intakes based on 3DDD (*r* = 0.241, *p* = 0.017) (Table A1).

Median energy intake for the study participants was 7481 (6334, 9104) KJ per day. The median daily intake of selected food groups was 1.8 (1.1, 2.7) serves of bread, 0.2 (0.0, 0.5) serves of fish and 1.4 (1.0, 2.1) serves of dairy; 24 participants (25%) consumed ≤1 serve of bread per day. The median estimated iodine intake based on 24-h urine for participants consuming <1 serve of bread daily (119 (70, 168) µg/day) appeared lower than those consuming ≥1 serves of bread daily (145 (102, 173) µg/day), although this was not significant. Based on dietary assessment the median estimated iodine intake for women consuming <1 serve of bread daily (52 (43, 74) µg/day) was significantly lower than those consuming ≥1 serves of bread daily (85 (68, 107) µg/day; *p* < 0.001).

Daily serves of bread, fish and dairy were not associated with iodine excretion. Daily serves of fish consumed were weakly associated with selenium excretion (*r* = 0.292, *p* = 0.004) as µg/L and (*r* = 0.206, *p* = 0.043) as µg/day. Iodine from 3DDD had a moderate correlation with serves of bread (*r* = 0.555, *p* < 0.001). There was a weak association between serves of fish and selenium from 3DDD (*r* = 0.216, *p* = 0.034). Body weight and BMI were not associated with any measure of iodine or selenium, however body weight was weakly associated with creatinine excretion per day (*r* = 0.228, *p* = 0.025) (Table A1).

## 4. Discussion

Despite the mandatory fortification of bread with iodised salt this group of postmenopausal women were defined as iodine deficient using both WHO and EAR cut-point definitions. The median UIC in the current study population (57 µg/L) is only slightly higher than that found prior to the mandatory fortification of bread in women aged 51–70 years in the 2008/9 New Zealand Adult Nutrition Survey (53 µg/L; 55 for men µg/L and 50 µg/L for women) [24]. Further 39% had a UIC below 50 µg/L, nearly double the 20% accepted by the WHO for a population regarded as iodine sufficient. Zimmerman and Andersson [8] advocate that the current UIC cut-off for adults is too high, suggesting a cut-off of 60–70 µg/L in adults; even using this more conservative cut-off would still classify the current study population as mildly deficient. However, the WHO definition of population iodine status is based on a spot sample not 24-h urine sample collected in the present study, thus the results need to be interpreted with caution.

The median UIC of the current study participants (57 µg/L with 39% < 50 µg/L) is slightly lower than seen in two previous New Zealand studies post-fortification which determined UIC in 24-h urine samples; with a higher percentage below 50 µg/L. A 2010 study in Palmerston North found women of childbearing age (*n* = 50) had a median UIC of 65 µg/L and 30% with UIC < 50 µg/L [25]. A 2012 New Zealand study in Dunedin and Wellington found women aged 45–64 years (*n* = 59) had a median UIC of 69 (42, 105) µg/L, with 32% with a UIC < 50 µg/L [23]. Two further recent New Zealand studies have determined UIC in spot samples. The most recent NZ data (2014/2015) from a representative population found a median UIC of 81 µg/L with 31% < 50 µg/L in women aged 51–70 years [26]. A 2014 study of older adults in long-term residential care found the women had a median UIC of 71 µg/L with 30% < 50 µg/L [27]. The high percentage of participants in the current study with low intakes is concerning.

In the present study the median urinary iodine excretion (UIE) was 124 µg/day. When this was converted to dietary intake (based on 90% of iodine excreted in the urine) the median intake was 138 µg/day which was below the RDI with 25% of the group below the EAR. Only two previous post-fortification studies have measured UIE in New Zealand women. Women of childbearing-age in Palmerston North (*n* = 50) had a median UIE of 117 µg/day, and estimated intake of 130 µg/day [25]; women aged 45–64 years from Dunedin and Wellington (*n* = 59) had a median UIE of 126 µg/day which equates to an intake of 140 µg/day [23]. The median iodine intakes from these three studies are below the RDI, and in the current study 25% had intakes less than the EAR, which suggests dietary inadequacy. One of the issues of looking solely at median intake is that this may appear sufficient at a population level, but there could still be a significant proportion of the population with inadequate intakes. Previous studies have found that NZ women consume an average of 2–3 slices of bread per day [23,28,29], this is similar to the current study where the median intake was 1.8 slices per day. However in the current study there are a significant proportion with low bread intake, 25% were consuming ≤1 slice per day. It is important that we investigate whether those who have low intakes of bread containing iodised salt either due to exclusion of bread or consuming organic bread are at risk of iodine deficiency.

The estimated median iodine intake from the 3DDD (excluding iodised salt) in the present study (79 µg/day) was greater than the mean dietary exposure estimated prior to mandatory fortification (excluding iodised salt) in the 2009 New Zealand Total Diet Survey for women aged over 25 years (63 µg/day) [30] and similar to the median intake (80 µg/day) post fortification estimated in NZ women aged 45–64 years using an FFQ (excluding iodised salt) [23]. Iodised salt has been reported to be used by 87% of women aged 51–70 years [24], however it is difficult to quantify the exact amount of discretionary salt (and hence iodine) added to food. Edmonds et al. [23] estimated daily use of 1 g of discretionary iodised salt equating to 48 µg iodine; adding this amount to the median 3DDD intake would yield 127 µg/day, which is still below the 138 µg/day of iodine based on UIE. Although it should be noted that in the Edmonds study the daily iodine intake from food plus iodised salt (126 µg/day) was also lower than the iodine intake estimated from UIE (138 µg/day) for women aged 45–64 years).

In the current study, estimated median selenium intakes were 45–50 µg/day, below the RDI (60 µg/day) and the mean dietary selenium exposure in women aged over 25 years (56 µg/day) in the 2009 New Zealand Total Diet Survey [30]. Estimated intakes were slightly higher from urine than 3DDD, with between 49%–55% below the EAR and 11% below the 30 µg/day suggested for adequate deiodinase function [12], which could compromise thyroid function. Current study intakes were comparable with median selenium intakes for women aged 51–70 years (47.0 µg/day) in the 2008/9 New Zealand Adult Nutrition Survey, where 55% were estimated to have an inadequate intake [24]. 

Collecting 24-h urine samples requires motivated participants and is not practical for all populations or large studies. Spot samples, although routinely collected, are complicated by variation in hydration status; iodine:creatinine ratio can correct for urine volume and is suggested to be a better measure of status than UIC alone [31]. Daily creatinine excretion is stable with adequate protein intake, although at low intakes it varies [31]. Low protein intakes are unlikely to be a problem in New Zealand, in the 2008/9 Adult Nutrition Survey only 1.5% of women aged 51–70 years were estimated to have an inadequate protein intake [24]. There are no published reference values for iodine:creatinine ratio however, a previous New Zealand study prior to mandatory fortification found a mean iodine:creatinine ratio in women of 45.9 µg/g [32], much lower than the current study (median 114.7 µg/g). Of interest, the earlier NZ study found significant associations between iodine and body weight [32]; this was not found in the current study, although the previous study participants were younger (18–58 years). Thyroid stimulating hormone (TSH), T4 and T3 are easily measured in blood samples, however deficient populations have concentrations within normal reference ranges so these are not sensitive measures of iodine status [9]. Thyroglobulin, a marker of thyroid hormone turnover, is a potential biomarker for iodine deficiency and can be measured in dried blood spot samples, however further work is required to validate methods and establish reference values [33].

Median energy intake for the study participants was 7481 (6334, 9104) KJ per day which was similar that seen in women aged 50–71 years of age in the 2008/9 New Zealand Adult Nutrition Survey (7071; 6777, 7385 KJ/day). Under or over-reporting is a concern for dietary assessment, however as energy expenditure was not recorded we were unable to determine if participants had misreported dietary intake. However, median estimated selenium intakes were not significantly different (*p* = 0.75) based on both dietary assessment (45 µg/day) and 24-h urine excretion (50 µg/day). If there had been significant underreporting we might expect selenium intake based on dietary assessment to be lower. The median selenium:creatinine ratio in the current study of women in the North Island of New Zealand was 26.5 µg/g, higher than seen in an earlier study in New Zealand’s South Island (12.5 µg/g) [32]; selenium intake is typically lower in the South Island where bread is made from local wheat compared to the North Island where bread is manufactured from wheat imported from Australia which has higher levels of selenium [4]. Again the earlier study found significant associations between selenium excretion and both body weight and BMI [32], these associations were not found in the present study. Determining selenium concentrations in blood (whole, plasma or erythrocyte), plasma Selenium protein P or GPx activity in blood (whole, plasma or platelet) are considered more reliable markers of selenium status [34]. However, urinary selenium excretion is associated with both plasma selenium and dietary intake in populations with low selenium intake [11]. A limitation of the current study is not measuring selenium or GPx activity in blood, however determining daily urinary selenium excretion serves as a proxy measure for selenium intake, and the current study indicates the need for further research in this population.

There is considerable daily variation in both iodine and selenium intake, however in this study one single 24-h urine sample was measured. Zimmerman and Andersson [8] recommend, when using the EAR cut-point method for iodine, collecting multiple samples in a subset of the population to account for individual variation. However, as this study represents baseline data from an intervention trial, repeat samples may have been influenced by the intervention and not represent habitual intake. The current study is not representative of the New Zealand population as the participants are self-selecting and do not reflect the national ethnic distribution. Women who volunteer for such studies are potentially more motivated towards a healthy lifestyle than the general population; also women with chronic diseases were excluded from the study. However, it is concerning many of these healthy individuals had low intake of selenium and iodine, which could compromise thyroid function.

## 5. Conclusions

In this study population iodine intakes have improved subsequent to the mandatory fortification of bread with iodised salt, compared to national New Zealand data prior to fortification. However the population is still classified as iodine deficient and 25% had estimated intakes below the EAR and 39% had a UIC < 50 µg/L. Selenium intakes are now more marginal than iodine intakes in this group, with 49%–55% below the EAR and 11% at risk of compromised thyroid function due to extremely low selenium intake. The current WHO cut-offs to determine population iodine status using UIC for spot urine samples may not be appropriate for adults and alternative biomarkers are required. Collection of 24-h urine samples is not feasible for large studies, thus the use of creatinine: iodine ratio requires further investigation. Alternatively blood based biomarkers such as thyroglobulin for iodine and GPx activity for selenium status need to be determined amongst postmenopausal women in New Zealand to investigate current status. More comprehensive studies are required throughout New Zealand to examine the effectiveness of mandatory fortification, especially amongst those with low intakes of iodised bread, and also to investigate if current intakes of iodine and selenium affect thyroid function and health. If further studies confirm these findings, then alternative approaches will need to be identified for low consumers of iodine and selenium.

## Figures and Tables

**Table 1:** Iodine, selenium and creatinine in 24-h urine samples from postmenopausal women.

*n* = 97	Median	Q1, Q3
Urine volume (L)	2.05	1.68, 2.50
Urinary iodine concentration (UIC) µg/L	57	41, 78
Measured 24-h urinary iodine µg/day	124	90, 155
Urinary selenium µg/L	14	10, 20
Measured 24-h urinary selenium µg/day	28	20, 39
Urinary creatinine g/L	0.48	0.38, 0.67
Urinary creatinine g/day	1.05	0.85, 1.25
Iodine:creatinine µg/g	115	84, 156
Selenium:creatinine µg/g	27	20, 40

**Table 2:** Estimated iodine and selenium intakes based on 24-h urine excretion and dietary assessment in postmenopausal women.

Nutrient intake (*n* = 97)	Iodine	Selenium
Estimated intake; median (Q1, Q3)		
^a^ Based on 24-h urine, µg/day	138 (100, 72) ^d^	50 (36, 71)
^b^ Based on 3DDD (without iodized) salt, µg/day	79 (55, 97) ^d^	45 (36, 68)
^c^ Below EAR (*n*, %)		
^a^ Based on 24-h urine, µg/day	24 (25)	48 (49)
^b^ Based on 3DDD (without iodized) salt, µg/day	74 (76)	53 (55)
Below 30 µg/day selenium intake (*n*, %)		
^a^ Based on 24-h urine, µg/day	-	11 (11)
^b^ Based on 3DDD (without iodized) salt, µg/day	-	11 (11)

^a^ based on urinary excretion of ingested intake of 90% for iodine and 55% for selenium; ^b^ 3DDD = three day diet diary; ^c^ EAR = Estimated Average Requirement; ^d^ *p* < 0.001 (Wilcoxon ranked sign test).

## Appendix A

**Table A1:** Correlation matrix for iodine, selenium and creatinine in 24-h urine samples in postmenopausal women using Spearman’s rho.

*n* = 97		Urine Volume L	Urine Iodine µg/L	Urine Iodine µg/Day	Urine Selenium µg/L	Urine Selenium µg/Day	Urine Creatinine g/L	Urine Creatinine g/Day	Iodine: Creatinine Ratio µg/g	Selenium: Creatinine Ratio µg/g	Iodine 3DDD
Urine Iodine µg/L	*r*	−0.502									
	*p*	<0.001									
Urine Iodine µg/day	*r*		0.757								
	*p*	ns	<0.001								
Urine Selenium µg/L	*r*	−0.464	0.509	0.263							
	*p*	<0.001	<0.001	0.009							
Urine Selenium µg/day	*r*			0.385	0.758						
	*p*	ns	ns	<0.001	<0.001						
Urine Creatinine g/L	*r*	−0.675	0.571		0.604						
	*p*	<0.001	<0.001	ns	<0.001	ns					
Urine Creatinine g/day	*r*		0.298	0.278	0.427	0.391	0.711				
	*p*	ns	0.003	0.006	<0.001	<0.001	<0.001				
Iodine:Creatinine ratio µg/g	*r*		0.478	0.714			−0.351	−0.375			
	*p*	ns	<0.001	<0.001	ns	ns	<0.001	<0.001			
Selenium:Creatinine ratio µg/g	*r*			0.200	0.475	0.706	−0.312	−0.256	0.398		
	*p*	ns	ns	0.049	<0.001	<0.001	<0.001	0.012	<0.001		
Iodine 3DDD	*r*			0.242					0.241		
	*p*	ns	ns	0.017	ns	ns	ns	ns	0.018	ns	
Selenium 3DDD	*r*				0.216	0.329				0.241	0.289
	*p*	ns	ns	ns	0.033	0.001	ns	ns	ns	0.017	0.004

3DDD = three day diet diary.
